# Vietnamese non-English-major students’ motivation to learn English: from activity theory perspective

**DOI:** 10.1016/j.heliyon.2021.e06819

**Published:** 2021-04-22

**Authors:** Son Van Nguyen, Anita Habók

**Affiliations:** a Doctoral School of Education, University of Szeged, Hungary; b Institute of Education, University of Szeged, Hungary

**Keywords:** Motivation, Vietnam, Activity theory, Motivational intensity, Non-English-major students

## Abstract

The purpose of this study was to investigate Vietnamese non-English majors' motivation to learn English as a foreign language (EFL) based on the activity theory perspective. The participants included 1,565 students with at least one semester of university-level English, of whom 13 participated in the semi-structured interviews. The data was collected using the five-point Likert scale motivation and desire surveys with 16 items and individual face-to-face interviews. The results from the quantitative and qualitative strands indicated that the participants were highly motivated to learn English. The sources of such motivation included obtaining a good job in the future, achieving success in academic studies, maintaining effective communications with foreigners, having personal enjoyment, and being influenced by other people. In addition, they were not only more internally (rather than externally) motivated, but they also demonstrated strong motivational intensity to learn English and enhance their language competence. Moreover, there was a strong positive relationship between internal motivation and motivational intensity, whereas there was a weak positive correlation between external motivation and motivational intensity. Activity theory was used as a lens to elaborate on the discussion of learners’ motivation in this study. The findings of the current study can be used by stakeholders, such as EFL educators, decision-makers and curriculum developers, to understand more about their students in terms of psychological issues and to design appropriate programs that can increase their learning motivation.

## 1 Introduction

Since English has become the international communicative language, people throughout the world are more inclined to learn the language. In this regard, following the adoption of *Doi Moi* (i.e., open-door economic and social policies) in 1986, the Vietnamese government has actively promoted English language education and conducted various educational reforms. One such reform aimed to enhance the quality of English language teaching and learning in the national education system from 2008 to 2020 to foster a competitive economy with a competent labor force (Ministry of Education and Training, 2008). However, although a wide range of English programs are being offered by English language centers (Lam and Albright, 2018), the level of English proficiency, especially among non-English majors, is still relatively low and uneven (Trinh and Mai, 2018). Arguably, this can be attributed to their low motivation to learn English (Ngo et al., 2017).

In general, motivation is considered an important and contributing factor in the language learning process. However, the research on the motivation to learn English in the context of Vietnam has been limited, with a scarcity of studies on non-English majors, who constitute the main portion of the English as a foreign language (EFL) population in the country (Nguyen and Habók, 2020). Therefore, based on the activity theory perspective, the purpose of this study is to examine the motivational orientation and intensity to learn English among a sample of non-English majors at seven public universities in Vietnam.

## 2 Theoretical background

### 2.1 Motivational orientation

The motivation to learn a second/foreign language (L2) is a complex construct and one of the most important factors for determining students' success in learning (Bradford, 2007; Sahril and Weda, 2018). Previous research has shown that there is a strong relationship between motivation and L2 achievement (Polat, 2011). In the seminal study on the role of motivation in L2 learning by Gardner and Lambert (1959), two types of motivation were identified: (1) integrative motivation and (2) instrumental motivation. The former refers to the desire to learn more about “the language group or to meet different people”, whereas the latter signifies a “more utilitarian value of linguistic achievement” (Gardner and Lambert, 1959, p. 192). In other words, integratively motivated students have a desire to affiliate within the target community. For example, students with integrative motivation prefer to learn English so that they can understand well in addition to knowing more about English-speaking people, and integrate into the English-speaking communities such as those in the United States (US), the United Kingdom (UK), Australia, Canada, and New Zealand. Meanwhile, instrumentally motivated students wish to learn English due to a practical reason such as having a better job opportunity or entering colleges. However, in countries where daily communications with native English speakers are not common and language learning is mostly confined to classroom settings, students lack opportunities to identify with those L2 groups (Yashima, 2002). Additionally, the context of today's world encourages us to assume an international posture that includes an “interest in foreign or international affairs, a willingness to go overseas to stay or work, the readiness to interact with intercultural partners, and, one hopes, an openness or a non-ethnocentric attitude towards different cultures, among others” (Yashima, 2002, p. 57). This also includes those with the instrumental motivation to succeed in certain objectives such as obtaining better employment.

According to the self-determination theory developed by Deci and Ryan (1985), motivation can be categorised into two types: (1) intrinsic motivation and (2) extrinsic motivation. When people are intrinsically motivated, they pursue an activity “in the absence of a reward contingency or control” since they find it “interesting and fun” (Deci, 1980, p. 34). Extrinsic motivation refers to any motivational orientation that is regulated by some instrumental means such as a monetary reward or a good job. There are also three sub-types of extrinsic motivation: (1) external regulation for gaining rewards or avoiding punishment; (2) introjected regulation for avoiding guilty feelings or self-aggrandising; and (3) identified regulation for consistency with what people value. In general, these sub-types are self-determined in ascending order.

Brown (2000) elaborated on the two types of motivation and established a framework of motivation (see Table 1). However, this framework does not explain all types of motivation.

**Table 1:** Motivational orientations by Brown (2000, p. 175), reprinted from Principles of language learning and teaching, Brown H. D., Page 175, Copyright (2000), with permission from Douglas Brown.

	Intrinsic	Extrinsic
**Integrative**	L2 learner wishes to integrate with L2 culture (e.g., for immigration or marriage)	Someone else wishes the L2 learner to know the L2 for integrative reasons (e.g., Japanese parents send kids to Japanese-language school)
**Instrumental**	L2 learner wishes to achieve goals utilizing L2 (e.g., for a career)	External power wants L2 learner to learn L2 (e.g., corporation sends Japanese businessman to U.S. for language training)

Kyriacou and Kobori (1998) reviewed the related literature and found three main sources of motivation to learn L2: (1) intrinsic reasons, which explicate “an interest in and enjoyment of learning and using a foreign language”; (2) instrumental reasons, which delineate “the uses and benefits that can follow proficiency in a foreign language such as improved career prospects and access to higher education”; and (3) integrative reasons, which emphasise “the way expertise in a foreign language can enable the user to participate in the culture of another region or country and interact with other people” (p. 345).

In the Taiwanese context, Hsu (2005) made distinctions between internal and external motivation. For instance, L2 learners are internally motivated by their own sake, whereas they are externally motivated due to outside pressure (e.g., parents, teachers or examinations).

Dörnyei (2005) proposed the L2 Motivational Self System, which included three components: (1) the ideal L2 self; (2) the ought-to L2 self; and (3) the L2 learning experience. The ideal L2 self or the ideal self-image emphasises a desire to use L2 competently, whereas the ought-to L2 self refers to “attributes that one believes one ought to possess (i.e., various duties, obligations or responsibilities) in order to avoid possible negative outcomes” (Dörnyei, 2005, p. 106). As for the third component, it focuses on “situation-specific motives related to the immediate learning environment and experience” (Dörnyei, 2005, p. 106).

In a comparative study, Csizér and Kormos (2009) explored the aforementioned motivational self-system among Hungarian EFL learners, including secondary school students and non-English majors. They suggested that the factors determining the self-concept consist of international posture, knowledge orientation, language learning experience, and parental encouragement. Notably, since such encouragement was the only factor that significantly affected the ought-to L2 self, it was found to be socially constructed.

In another comparative study among Japanese, Chinese, and Iranian EFL learners, Taguchi et al. (2009) concluded that integrativeness can be re-interpreted as the ideal L2 self, and instrumentally, it can be classified into two types: (1) promotional tendency, which is related to the ideal L2 self and (2) preventional tendency, which is associated with the ought-to L2 self. For example, they stated that studying abroad is promotional if an individual desires to go overseas, but it becomes preventional if he/she is required to do so by a company or organization.

Overall, this literature review not only provides us with a theoretical background on motivation, but it also shows that L2 motivation is a frequently discussed topic that requires further investigation from different contexts and perspectives (Dörnyei & Ushioda, 2013). In this particular way, the present study investigates non-English-major students’ motivation to learn English in Vietnam from the activity theory perspective.

### 2.2 Activity theory

**Figure 1:**
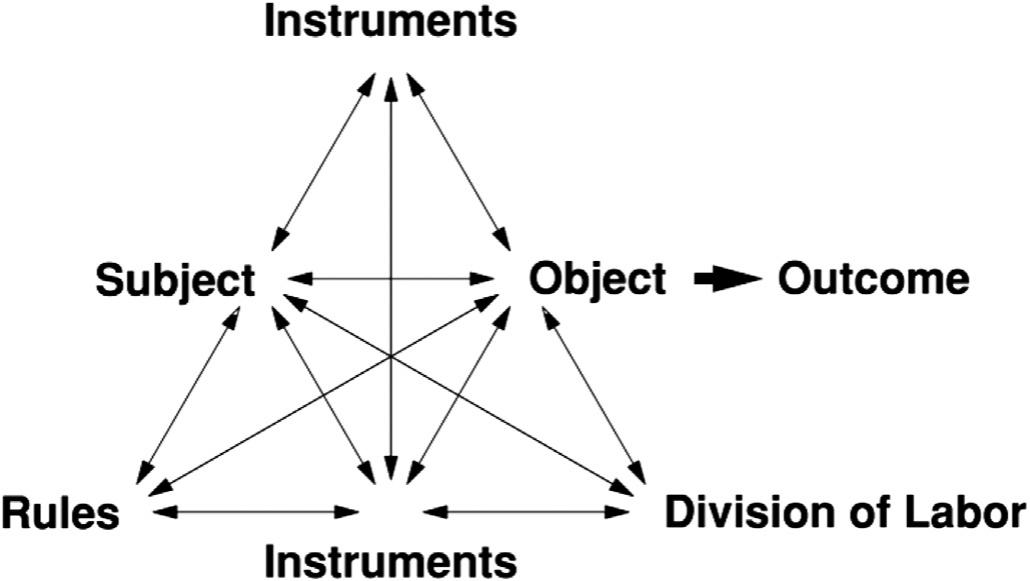
A model of activity (Engeström, 1987, p. 78), reprinted from Learning by Expanding: An activity-theoretical approach to developmental research, Engeström Y., Page 78, Copyright (1987), with permission from Yrjö Engeström.

Contributing to the analysis of the motivational processes in language learning (Ushioda, 2007), activity theory elaborates on different concepts from socio-cultural theory and particularly focuses on the motivational dimension of human activities (Allen, 2010). To describe an activity, Leontiev (1978) employed three elements (i.e., subject, object, and tools) that operate on three levels (i.e., collective activity, individual or group action, and automatic operation). Engeström (1987) elaborated on Leontiev (1978)'s framework and specified six components of an activity, including subject, object, tools and artefacts, community, rules, and the division of labor. These six components still operate on the same three levels defined by Leontiev (1978). Subsequently, the relationship among the components is delineated as follows (see more in Blin, 2004). The subject does not work in isolation, but he/she is a member of a community that includes many other participants with the same object. Moreover, tools and artefacts as well as rules mediate the relationship between the subject and the object, while the relationship between the community and the object is mediated by the division of labor, which “encapsulates both the horizontal distribution of tasks between peers and the vertical distribution of power between participants” (Blin, 2004, p. 383) (see Figure 1).

The present study argues that L2 learners’ motivation is highly relevant to activity theory (see more in Gedera, 2016; Kim, 2013). That relevance is demonstrated as follows:

• Subject: non-English-major students as the primary focus of study.
• Object: the goals pursued by the students.
• Tools and artefacts: the instruments used to learn English (e.g., books, computers, and the Internet).
• Community: teachers, peers, friends, and family members.
• Rules: norms, conventions, and regulations.
• Division of labor: the shared responsibilities among the community.

It is important to note that if there is no tension among these components, then motivation will either increase or at least be maintained (Kim, 2013). Those components above show an inter-relatedness, which facilitates the understanding of EFL learners’ motivation that sometimes increases and decreases in the same environment (Kim, 2013).

**Figure 2:**
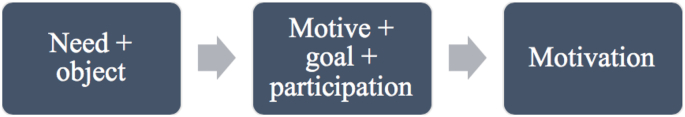
The development of motivation from activity theory perspective (Kim, 2007), reprinted from Second language learning motivation from an activity theory perspective: Longitudinal case studies of Korean ESL students and recent immigrants in Toronto, Kim T. Y., Copyright (2007), with permission from Tae-Young Kim.

From the activity theory perspective, each human activity is motivated by a specific biological or culturally constructed need that becomes a motive when directed by an object (Allen, 2010; Lompscher, 1999). The development of motivation to learn languages is illustrated in Figure 2. The motive culturally, psychologically, or institutionally guides the activity toward the object. The activity is represented by goal-oriented actions, and goals explicate a person's engagement in activities (Allen, 2010; Kim, 2007). A motive and a goal are aligned with participation in a new community of practice and result in motivation for language learning (Allen, 2010; Kim, 2007; Lave and Wenger, 2007).

## 3 Empirical background

Previous research on the motivation to learn a foreign language has been conducted for several decades. In particular, numerous studies have examined the motivation of university students in different contexts, mainly in non-native speaking countries. This part of the review focused on the studies investigating non-English-major students' motivation to learn English. First, in Taiwan, Warden and Lin (2000) used questionnaires to survey 442 non-English majors and found that the majority of the students lacked integrative motivation, but had strong instrumental motivation and required motivation (e.g., to pass an examination). Second, in Bradford's (2007) study conducted in Indonesia, the sample of university students (N = 168) showed a high level of motivation due to pragmatic reasons (e.g., money or well-paid jobs), but a low level of integrative motivation to identify with English native speakers. Third, in China, the sample of third-year university students (N = 202) in Liu (2007)'s quantitative study were highly motivated to learn English and were more instrumentally motivated than integratively motivated. Finally, in Yemen, Al-Tamimi and Shuib's (2009) mixed study, which included a survey of 81 students majoring in petroleum engineering, followed by semi-structured interviews with 10 students, found that instrumental motivation had a greater impact on the students' learning than integrative motivation.

Meanwhile, limited studies have focused on the motivation of non-English-major students in Vietnam. First, Phan's (2010) qualitative study of 10 technical-English-major students (which included interviews and examinations of their weekly diaries) found that they were intrinsically motivated to learn English, but it could change due to other types of motivation such as external and instrumental motivation. Second, Vu and Rochelle (2015) administered questionnaires to 193 students, of whom 45 were interviewed. They found that the students were slightly more instrumentally motivated than integratively motivated, with no significant difference between the males and the females. Third, using the expectancy-value model, Truong (2016) triangulated his mixed-method study with surveys of 1,207 first-year, non-English-major students, individual interviews with nine lecturers, and focus-group interviews with 72 students. He found that most of the participants were motivated to learn English and achieve a certain level of oral English competency to obtain good jobs in the future. Fourthly, Ngo et al. (2017) compared the motivation to learn English between English majors and non-English majors by using questionnaires. They found three types of motivation demonstrated by the non-English majors: (1) a moderate level of obligation; (2) a high level of professional development; and (3) a moderate level of intrinsic motivation. Finally, Nguyen (2019) conducted a purely quantitative study on second-year university students using survey questionnaires and indicated that the participants were highly motivated to learn English and tended to demonstrate instrumental motivation. It is notable that no studies have been found to apply activity theory to investigate motivation in the context of Vietnam. Moreover, there is a need for more studies on motivation to learn English from different perspectives with an emphasis on non-major students.

Overall, the previous studies share two common points. First, methodologically, the studies employed questionnaires, interviews, and even examinations of weekly diaries to collect the data. Second, the studies were carried out at a single institution. Since Rifkin (2000) postulated that the local conditions of a single institution can presumably limit the research results, the present study focused on more than one university.

Based on the lack of empirical research in this area, the present study aims to fill this gap by addressing the following questions:

(1) How motivated are non-English-major students to learn English?
(2) How can students' motivational intensity to learn English be described?
(3) Is there a relationship between motivation and motivational intensity?

## 4 Methodology

### 4.1 Context

Previous research has argued that contextual factors can have a direct influence on motivation (Coleman et al., 2007). Accordingly, motivation can be investigated through “the dynamic interaction between the learner and a complex system of social relations, cultural contexts and the learning environment” (Coleman et al., 2007, p. 247). Thus, it is necessary to explore the context of the research. In regard to the present study, the policymakers launched strategic programs (both international and nationally) to enhance English learning in Vietnam, due to globalization and internationalization (Phan, 2021). Moreover, the Vietnamese government cooperated with various international organizations such as the Association of Southeast Asian Nations (ASEAN), the World Trade Organization (WTO), and the Asia Pacific Economic Cooperation (APEC). The common point is that they used English as a communication tool. Moreover, one of the requirements from many employers is English proficiency, which is demonstrated through interviews and/or language certificates such as the Test of English for International Communication (TOEIC), or the International English Language Testing System (IELTS). Institutionally, the Vietnamese government adapted the Common European Framework of Reference (CEFR) as a standardized English proficiency measure. Accordingly, non-English-major students must achieve Level 3 (B1) in the adapted version of the CEFR upon graduation. Furthermore, each graduate must complete 10 credits of English, which includes three periods per week (on average) over three semesters. It is up to the universities’ decision-makers to ensure that the students are able to achieve the required English proficiency level.

### 4.2 Participants

Overall, the participants included 1,565 non-English-major students with at least one semester of university-level English from seven public universities in Hanoi, Vietnam, where we had contacts with their respective English departments. Those students whose mother tongue was Vietnamese were from 19 to 22 years old. The foreign language they knew was only English and their English proficiency varied from elementary to intermediate. Based on convenience sampling, we invited 20 out of the 1,565 participants, of whom 13 agreed to participate in the interview phase. Table 2 presents the background information of the survey participants, while the interviewees' profiles are presented in Table 3.

**Table 2:** Background information of the survey participants.

Gender (%)		Year of study (%)				Major (%)							Years spent studying English
Male	Female	2^nd^	3^rd^	4^th^	5^th^	IT	CE	E	EE	ME	L	Other	M = 11.7
62.2	37.8	62	23.7	11.9	2.4	21.7	7.9	11.8	16.5	12.2	12	17.9	SD = 1.4

Note: IT = Information Technology; CE = Civil Engineering; E = Economics; EE = Electrical and Electronic Engineering; ME = Mechanical Engineering; L = Law.

**Table 3:** Profiles of the interviewees.

Gender (%)		Year of study	Major (%)							Years spent studying English
Male	Female	2^nd^	IT	CE	E	EE	ME	L	MM	12

53.8	46.2		15.38	15.38	15.38	15.38	15.38	7.69	7.69	

Note: IT = Information Technology; CE = Civil Engineering; E = Economics; EE = Electrical and Electronic Engineering; ME = Mechanical Engineering; L = Law; MM = Multi-media. Engineering; L = Law.

### 4.3 Instruments

The data was obtained using the motivation and desire scales (Table 4) from the validated questionnaire (Nguyen and Habók, 2021) and employing the semi-structured interviews later.

**Table 4:** The items in the scales.

Statement	Strongly disagree	Disagree	Neutral	Agree	Strongly agree
Motivational orientation					
1. I learn English because I find it very interesting.					
2. I learn English so that I can communicate with people who can speak English.					
3. I learn English because it will help me to get a good job.					
4. I learn English because it will help me to be successful in my studies.					
5. I learn English because I want to pass exams.					
6. I learn English because it is a required course at my university.					
7. I learn English because I want to be as good at English as someone I know.					
8. I learn English because I want to please my family.					
Motivational intensity					
1. When it comes to English tasks, I work very carefully to make sure I understand everything.					
2. If I have any opportunities to use English outside class, I will use it most of the time and Vietnamese if necessary.					
3. If my teacher wanted someone to do an extra English assignment, I would definitely volunteer.					
4. I would like to have friends from English-speaking countries.					
5. If English were not taught at my university, I would try to obtain lessons in English somewhere else.					
6. During English classes, I would like to have as much English as possible used.					
7. If there were an English club at my university, I would be interested in joining.					
8. Considering how I learn English, I can honestly say that I just do enough to get along.					

The scales included part of the questionnaire developed by Nguyen and Habók (2021) with good psychometrics (α = 0.902; χ^2^ = 1633.966; d.f. = 367; χ^2^/d.f. = 4.45 < 5.0; p < 0.01; SRMR = 0.057 < 0.06; RMSEA = 0.047 < 0.05; RMS_theta = 0.104 < 0.12). The first scale (i.e., *motivational orientation*) was initially produced by Brown (2002) and adapted from Hsu (2005) and Swatevacharkul (2009). In this case, the factor analysis (FA) revealed two factors: (1) internal motivational orientation and (2) external motivational orientation (KMO = 0.884, p < 0.01, total variance explained (TVE) = 48%). The second scale (i.e., *motivational intensity*) was originally generated by Gardner (1985) and adapted from Hsu (2005). In this regard, the FA indicated only one factor referring to motivational intensity or the desire to learn English (KMO = 0.920, p < 0.01, TVE = 51.2%). Overall, the scales were psychometrically sound, with an internal consistency (i.e., Cronbach's alpha) of 0.782 and 0.796, respectively; a rho_A reliability of 0.790 and 0.821, respectively; and a composite reliability (CR) of 0.854 and 0.867, respectively). Based on the analyses, these scales were reliable (Cohen et al., 2018). There were eight items in the first scale in which items 1–4 belonged to *internal motivational orientation* and items 5–8 were in *external motivational orientation*. Furthermore, there were eight items in the second scale. All the items in the scales were designed on the basis of the five-point Likert scale. The students were asked to choose one of the following responses that best reflected their level of agreement: 1 = *strongly disagree*; 2 = *disagree*; 3 = *neutral*; 4 = *agree*; and 5 = *strongly agree*. After being translated from English with the support of back translation methods and experienced English language instructors, the final questionnaires delivered to the participants were totally in Vietnamese language so that they could fully understand the scales.

As for the semi-structured interviews, their purpose was to elicit more in-depth information regarding the students’ motivation to learn English. The 13 interviews were held individually and face-to-face, entirely in the Vietnamese language, with the protocol designed in advance. The main interview questions included: Do you like/dislike learning English and why? How long have you been learning English? What are the reasons that motivate you to learn English?, and How willing are you to learn English?

### 4.4 Data collection

After obtaining ethical approval from the institutional review board of the researchers' university (Doctoral School of Education, University of Szeged, Hungary) and the permission of the participating universities, we visited the various classrooms and explained the research aims, objectives, significance, methodology, and ethics. All of the participants were told that their responses would be completely confidential and only used for research purposes. The paper-and-pencil questionnaires were administered to 1,600 students after which 1,565 were deemed useable (98% response rate). In this case, 35 questionnaires were excluded because they were either incomplete or the respondents did not want to include their answers. All the questionnaires printed and collected were stored in a secure locked in our office at university. Both of us were the only people to have access to those resources which would be retained in five years after the research's completion.

Among the students, 13 from different universities were randomly selected for the interviews, which were audiotaped with their consent. Each interview was approximately 20 minutes in length, which provided us with more insights into the student's thoughts and opinions.

### 4.5 Data analysis

Overall, we used a convergent parallel analysis design. Specifically, the quantitative and qualitative data were analyzed separately before the results were combined for explanations and discussions of the findings (see more in Creswell and Plano Clark, 2017). As for the former, we used the Statistical Package for Social Science (SPSS; Version 24) for the descriptive and inferential statistics. As for the latter, we transcribed the audio-recorded interviews, translated the transcripts into English, and had English language teaching (ELT) experts proofread them. Then, the final English version was entered into the ATLAS.ti software for recurrent themes that illustrated *motivational orientation* and *motivational intensity*. Both of us completed the coding process separately and then checked for consistency. We reached approximately 90% of agreement on coding results, which spoke for a high inter-rater reliability. To protect the students’ identities, they were each given a code, ranging from S1 to S13 before the coding of qualitative interview data was done. Afterwards, the two strands of data were combined, compared, and contrasted on the basis of themes (e.g., orientations of motivation and motivational intensity) to examine consistencies as well as discrepancies in two datasets and to reach proper conclusions regarding the research questions.

## 5 Results

### 5.1 Motivational orientation

Based on the analysis of the scale data, the majority of the participants selected the *agree* and *strongly agree* options regarding internal motivation (IM), whereas the percentages of the *disagree* and *strongly disagree* options regarding external motivation (EM) were higher than those in the internal counterpart (see Table 5).

**Table 5:** Descriptive statistics on students’ motivation to learn English.

Statements	M	Sd	SD %	D %	N %	A %	SA %
1. I learn English because I find it very interesting.	3.66	0.93	2.0	7.5	31.6	40.4	18.5
2. I learn English so that I can communicate with people who can speak English.	4.04	0.86	0.9	4.4	16.4	46.5	31.8
3. I learn English because it will help me to get a good job.	4.47	0.69	0.3	0.6	7.5	35.3	56.4
4. I learn English because it will help me to be successful in my studies.	4.28	0.76	0.4	1.8	11.2	42.9	43.7
5. I learn English because I want to pass exams.	3.4	1.09	6.0	16.0	24.0	40.3	13.7
6. I learn English because it is a required course at my university.	2.92	1.19	13.7	24.3	28.7	23.1	10.2
7. I learn English because I want to be as good at English as someone I know.	3.6	1.08	3.8	12.8	25.6	35.4	22.4
8. I learn English because I want to please my family.	2.85	1.12	12.7	26.4	31.2	22.6	7.0

Note: M = Mean, Sd = Standard deviation, SD = strongly agree, D = disagree, N = neutral, A = agree, SA = strongly agree.

Moreover, the inferential statistics revealed that there was a significant difference in the scores between IM (M = 4.11, Sd = 0.53) and EM (M = 3.19, Sd = 0.75) (t (1564) = 39.38, p < 0.01) (see Table 6).

**Table 6:** A paired sample t-test between IM and EM.

IM		EM		*df*	*t*	*p*
M	Sd	M	Sd			
4.11	0.53	3.19	0.75	1564	39.38	.000

Note: p < 0.01.

The interviewees also stated that they were motivated by communicative purposes, their own interests, personal enjoyment, study and career path, and other people such as friends, family people, and teachers (see Table 7).

**Table 7:** Motivation to learn English among 13 interviewed students.

	Own interests	Communicative purposes	Study & career	Personal enjoyment	Other people
S1		x			x
S2	x		x		x
S3			x		
S4		x			x
S5	x	x	x		x
S6		x		x	
S7	x		x	x	x
S8		x	x		x
S9		x	x		
S10		x	x	x	x
S11	x	x	x	x	
S12	x	x			
S13		x	x	x	

Overall, the first four sources of motivation can be categorized into IM, whereas the last one belongs to EM. Regarding communicative purposes, 10 students concurred that they learned English because they wanted to communicate in English with foreigners. This was evident in the following illustrative quotes:

*I learn English for communicating with international friends......Yes, my*

*motivation is communication....I truly want to talk to them in English.* (S1)

*I want to learn English to improve my communicative skills in English.* (S4)

*I really love being able to talk with foreigners when I have chances, so that is why I learn English.* (S6)

There were also five students who regarded their interest in languages as their motivation. For example, S2 expressed the following:

*English is not an important compulsory subject, but I like it. It is not due to my parents, but it is simply my own interest. I find it very interesting.* (S2)

*I learn English because I find it very interesting and I will learn a lot of new things for my life from that language.* (S7)

Similarly, S12 postulated that her only reason for studying English was that she loved learning languages, although she admitted that it may be useful for her future job.

As for the motivation to learn English, five interviewees stated that one of their motives was personal enjoyment. This point is exemplified by the following quotes:

*My motivation to learn English was to listen to music and to play the guitar. If I am good at English, I will be able to get access to the international US-UK music and to understand more about my music idols.* (S10)

*I learn English because I would love to read English stories or watch movies in English. The feeling when I understand the points in stories or movies is so great.* (S13

Similarly, S6 wanted to listen to English music and watch English films, while S7's hobby was traveling.

Interestingly, one commonly shared motivator among the participants was their study and future career path. For instance, S13 was unsure whether she enjoyed learning English, but she confirmed that she learned the language for her future job:

*Mainly, I study English for my future career. Since my major is multimedia, most of the documents are translated from English. So, I want to read the materials in English. I also want to improve the quality of my job later on. If I have a high level of English, then I believe that there will be more opportunities to work with foreigners. Consequently, my salary and remuneration will be higher. Nowadays, English is something vital, rather than compulsory.* (S13)

*Learning English well will be advantageous to my future job. I believe that if I can use English proficiently, the likelihood that I will get a well-paid job will be definitely higher.* (S3)

*Currently, we are living in the era of globalization, so without English, it becomes difficult to apply for a good job. As a result, I learn English to fulfill the language requirements*. (S9)

Finally, some students were motivated by other people (both in reality and on the Internet) such as family members (e.g., S1, S7, and S10), friends (e.g., S2 and S5), and teachers (e.g., S8). In general, this influence was positive because no one stated that they learned English to please other people. For instance, S4 stated the following:

*During my first year at the university, I realised that many people could speak English fluently and I wanted to be like that.......As for the Internet, many vloggers and those on YouTube record themselves in English. This made me excited to learn the language.* (S4)

*One of my friends could speak English very well, which motivated me to learn English so that one day I could use English as confidently as her.* (S2)

One aspect that drew our attention was that although some of the students wanted to pass examinations, it was not their primary motivation. Notably, the majority of the interviewees presented several reasons why they wanted to learn English. For example, S11 learned English for four reasons: (1) his interests, (2) communicative purposes, (3) study and career, and (4) personal enjoyment. Meanwhile, S13 shared that the main reason was for better communication skills in English and ultimately a better job.

Overall, the statistical and thematic analyses showed that the students in our sample, despite being from different universities, had a high level of IM to learn English and a moderate level of EM. Their motivation primarily concentrated on career prospects, study opportunities, and communicative needs.

### 5.2 Motivational intensity

The results of the quantitative analysis showed that the students mainly selected the *neutral*, *agree* and *strongly agree* options, except for those in Item 8. Table 8 presents the findings regarding the *motivational intensity* (MI) of the participants.

**Table 8:** Descriptive statistics on motivational intensity.

Statements	M	Sd	SD %	D %	N %	A %	SA %
1. When it comes to English tasks, I work very carefully to make sure I understand everything.	3.62	0.84	0.8	7.0	36.4	41.3	14.5
2. If I have any opportunities to use English outside class, I will use it most of the time and Vietnamese if necessary.	3.45	0.94	1.5	13.5	38.0	33.2	14.1
3. If my teacher wanted someone to do an extra English assignment, I would definitely volunteer.	3.25	0.87	3.6	10.2	51.7	27.0	7.6
4. I would like to have friends from English-speaking countries.	3.92	0.86	1.0	4.5	22.3	46.1	26.2
5. If English were not taught at my university, I would try to obtain lessons in English somewhere else.	3.89	0.84	1.0	3.9	24.1	46.9	24.2
6. During English classes, I would like to have as much English as possible used.	3.83	0.84	1.0	3.5	28.7	45.0	21.8
7. If there were an English club at my university, I would be interested in joining.	3.12	0.94	4.5	17.6	47.9	21.7	8.4
8. Considering how I learn English, I can honestly say that I just do enough to get along.	2.73	1.08	13.5	30.5	30.8	20.3	4.9

Note: M = Mean, Sd = Standard deviation, SD = strongly agree, D = disagree, N = neutral, A = agree, SA = strongly agree.

Correlational analyses were also conducted to examine the relationship between IM and MI and between EM and MI. The results indicated a strong positive relationship between IM and MI (r = 0.51, p < 0.01) and a weak positive relationship between EM and MI (r = 0.06, p = 0.02) (see Tables 9 and 10). Moreover, after the coefficient of determination (r^2^) calculated, there was a covariance of 26.01% between IM and MI.

**Table 9:** Correlations between IM and MI.

		IM	MI
IM	Pearson correlation	1.000	.51∗∗
	Sig. (2-tailed)		.000
	N	1565	1565
MI	Pearson correlation	.51∗∗	1.000
	Sig. (2-tailed)	.000	
	N	1565	1565

∗∗ Correlation is significant at the 0.01 level.

**Table 10:** Correlations between EM and MI.

		EM	MI
EM	Pearson correlation	1.000	.06∗∗
	Sig. (2-tailed)		.020
	N	1565	1565
MI	Pearson correlation	.06∗∗	1.000
	Sig. (2-tailed)	.020	
	N	1565	1565

∗∗ Correlation is significant at the 0.05 level.

Despite the various reasons why they learned English, all of the interviewees stated that they would take opportunities to learn and improve their English such as trying to understand English tasks, having foreign friends, and using English both in and outside of class. For instance, S3 stated the following:

*For sure, I will take opportunities to learn English because class time is never sufficient. The time to learn English in secondary school, in particular, did not enable me to use the language well.* (S3)

*….Why not? Those opportunities to learn English will definitely facilitate my improvement in English proficiency. Also, they will bring me closer to the world in the way that I will be engaged in international communities.* (S11)

However, several students (i.e., S2, S7, S8, and S9) noticed that it sometimes depended on specific conditions. For example, typically, “there may be too many other things to do at home or in class. There are too many subjects with heavy workloads” (S7) . In addition, seven students stated that they would find a center to learn English if it were not taught at the university, while the other interviewees agreed that they would do more tasks if their teachers asked them to do so. In regard to the latter, S13 shared that although he did not regularly volunteer to do tasks, he never refused them because he knew that they were good for his academic studies. Moreover, eight participants denied that they only learned English to get along and acknowledged the role of English in globalization, as illustrated in the following:

*I particularly want to study English well. No, it is not just to cope with class tasks. It is for my future use of English.* (S7)

*I never think that I learn English merely to pass the courses or the exams. I myself know how important it is to my life and to the fourth industrial revolution.* (S6)

Finally, most of the interviewees were interested in participating in English learning communities, including clubs as chances to practice the language. For example, S1 said, “English club at my university aims to help students improve their English skills, which is good. I would like to attend the sharing sessions held by that club”. Moreover, S8 expressed his thought that “there is an English club at my university which brings so much fun to its members. I think I am keen on attending it as it is an effective way to practice the language in a fun way”. However, they did not find the activities interesting (e.g., S12) or the schedules did not fit into their daily routines (e.g., S10). More importantly, they could not practice English much inside or outside classrooms because it is not yet a second language, not many people speak it in daily life, and the students could not meet foreigners frequently to learn English on a regular basis (e.g., S2, S4, S7, and S9). This entailed the obstacle encountered by many students in this study. They claimed that their language instructors and peers who were Vietnamese, the course books, the supplementary materials, and the Internet became the paths that brought them to the world of English.

*The major barrier to English communications is that English is not used much in daily life. Seemingly, people prefer speaking Vietnamese to save time of expression. They do not speak English. The ways that I can be exposed to English are from textbooks, supplementary materials, and the Internet.* (S2)

*Well, we only work with books, our teacher and friends in class and the Internet at home. One pity is that there are not many opportunities to see and communicate with international friends regularly in Vietnam.* (S9)

To summarize, the participants in this Vietnamese sample showed a strong desire to learn English. That desire was expressed by the following activities: attending English classes under any circumstances, making friends with foreigners, volunteering to do more tasks, and using English as much as they could. It was also clear that they do not just learn English to get along. More notably, the relationship between IM and MI was relatively strong. Specifically, the more internally motivated the students were, the higher the level of MI they demonstrated.

## 6 Discussion

The purpose of this study was to investigate the motivation and desire to learn English among a sample of non-English-major students at seven public universities in Vietnam. The findings provided considerable evidence that the students were highly motivated to learn English. They also reported a high level of IM. For example, the students found the English language intriguing and they were strongly motivated to learn English to improve their academic studies, prepare for their future careers, communicate with foreigners, and satisfy their personal enjoyment. Moreover, the participants demonstrated a moderate level of EM. For instance, according to the interviews, other people inspired them to learn English such as friends, peers, someone on the Internet, teachers, and parents. Interestingly, although many students wanted to pass English examinations, they confirmed that it was not their main motivation. The students also disagreed that they learned English to please their family or to get along with the university requirements, which was consistent with the thematic data.

According to the theoretical background, those non-English majors have three reasons for learning English: (1) intrinsic motivation, (2) integrative motivation, and (3) instrumental motivation (see Kyriacou and Kobori, 1998). They also express their ideal L2 self (see more in Yashima, 2002; Dörnyei, 2005; Csizér and Kormos, 2009; Taguchi et al., 2009). Specifically, that ideal L2 self (i.e., aspirations, hopes, and wishes) motivates them to learn English because they find it interesting, they wish to communicate with foreigners, and more notably they believe that it will help them become successful in the future and will aid them in becoming more proficient in English like someone they know (Dörnyei, 2009). Meanwhile, they did not demonstrate the ought-to L2 self (see more in Cho, 2020; Dörnyei & Ryan, 2015; Tseng et al., 2020), as they indicated that they learned English not because of expectations, duties, obligations, or avoidance of negative outcomes. Arguably, their demonstration of L2 self was a positive sign because as a powerful motivator, the ideal L2 self reduces the difference between actual and ideal selves (Dörnyei and Chan, 2013).

Overall, the results of the present study differ from the preconceptions of previous research in which non-English-major students are not motivated to learn English or they learn English because it is compulsory (Tran and Baldauf, 2007). The reasons may be due to the students’ awareness of the importance of English in the era of the fourth industrial revolution and the integrated learning mode at universities that is different from the traditional language teaching approach (Oxford et al., 1994; Su, 2007). However, the findings are in line with those of previous studies (e.g., Al-Tamimi and Shuib, 2009; Bradford, 2007; Chairat, 2015; Chen et al., 2005; Köseoğlu, 2013; Liu, 2007; Liu and Huang, 2011; Ngo et al., 2017; Rahman, 2005; Vaezi, 2008; Vu and Rochelle, 2015; Warden and Lin, 2000) in which the main motivation among non-English-major students is professional development. Such motivation demonstrated by the students can be explained by the socio-cultural context in which English holds a dominant position (Le and Chen, 2018; Nguyen, 2017), which ultimately fosters cultural exchanges, overseas studies, and good jobs.

Regarding MI, due to the influence of the socio-cultural context in Vietnam aforementioned (see more in Le and Chen, 2018; Phan, 2021), the students in this study were fully aware of the significance of English. Accordingly, the students desired to achieve the following: learn English to make friends with English-speaking people, attend English classes under any circumstances, use English as much as possible, and volunteer for more tasks. It is noteworthy that IM and MI were strongly positively correlated, whereas the relationship between EM and MI was weak (see more in Ngo et al., 2017; Noels, 2001). Thus, a higher level of IM would probably lead to an increase in MI.

**Figure 3:**
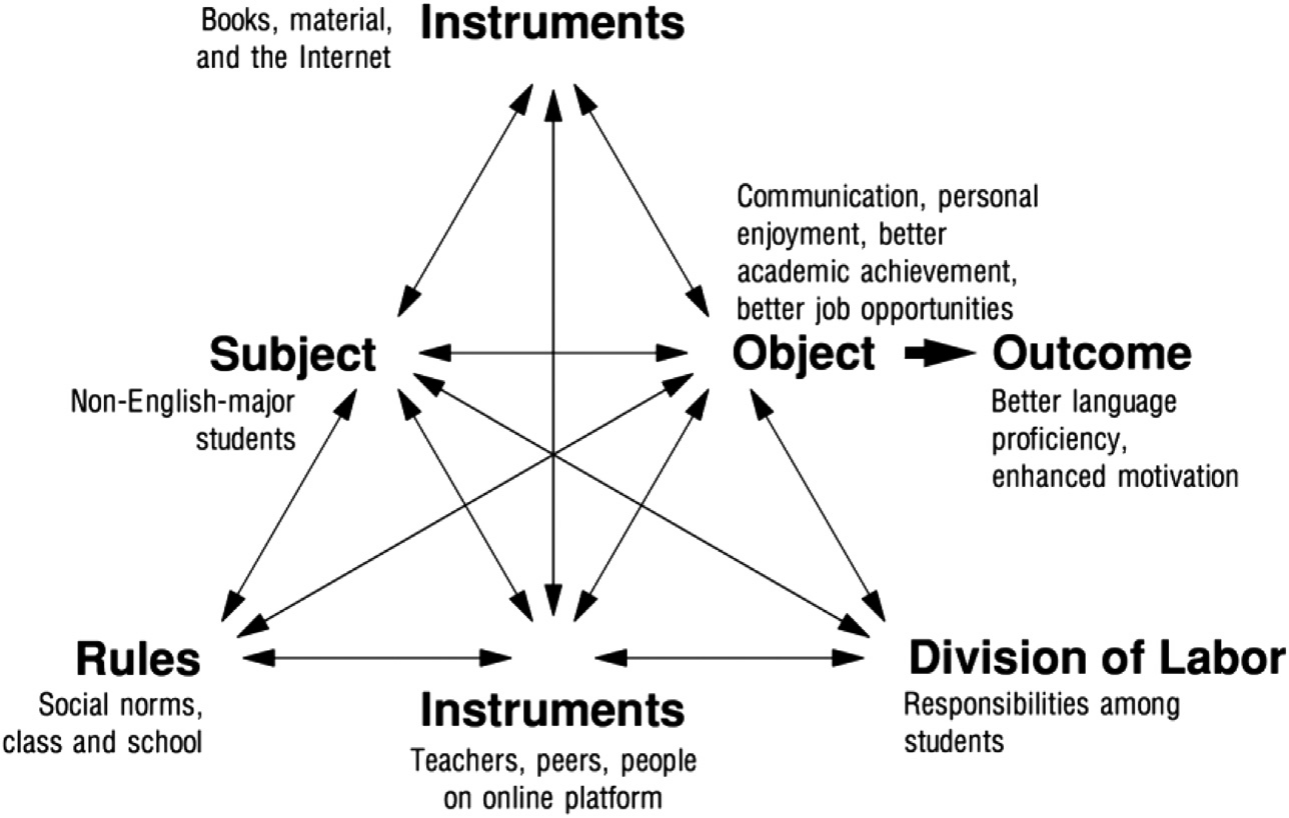
Students' motivation in activity system, reprinted from Learning by Expanding: An activity -theoretical approach to developmental research, Engeström Y., Page 78, Copyright (1987), with permission from Yrjö Engeström.

From the activity theory perspective, the students' motivation in the current study is presented in Figure 3. The non-English majors in this study (i.e., the subject) were learning English not because it was required, but because they were working toward certain goals such as language communication, personal enjoyment, better academic studies, and better job prospects (i.e., the object). These students were also members of a language learning community, which included real people (e.g., teachers and peers) and those in virtual platforms such as Facebook or YouTube. Moreover, in order for them to learn English, books, materials, and the Internet were necessary (i.e., tools and artefacts) although there was a lack of real communications with foreigners and the students had to conform to social norms and school regulations based on the work shared among the community members. In this regard, the tools and norms interceded the relationship between the students and their goals, which entailed helping them achieve their goals. Finally, the community promoted the students' achievement of goals by defining the division of labor. For example, the teachers’ warmth, friendliness, and supportiveness motivated the students to learn more effectively and efficiently. According to the activity theory, there are interactions and interrelations between the students (i.e., subject) and the objects which they act on and modify or produce (Lompscher, 1999). The relationship between the participants and their objects is mediated by materials and the Internet, the relationship between those students and the learning community is mediated by class and school rules or regulations, and the relationship between the objects and the community is interceded by the division of labor (see more in Heo and Lee, 2013; Kuutti, 1995). The relationships among the components in the activity system are illustrated in Figure 3.

**Figure 4:**
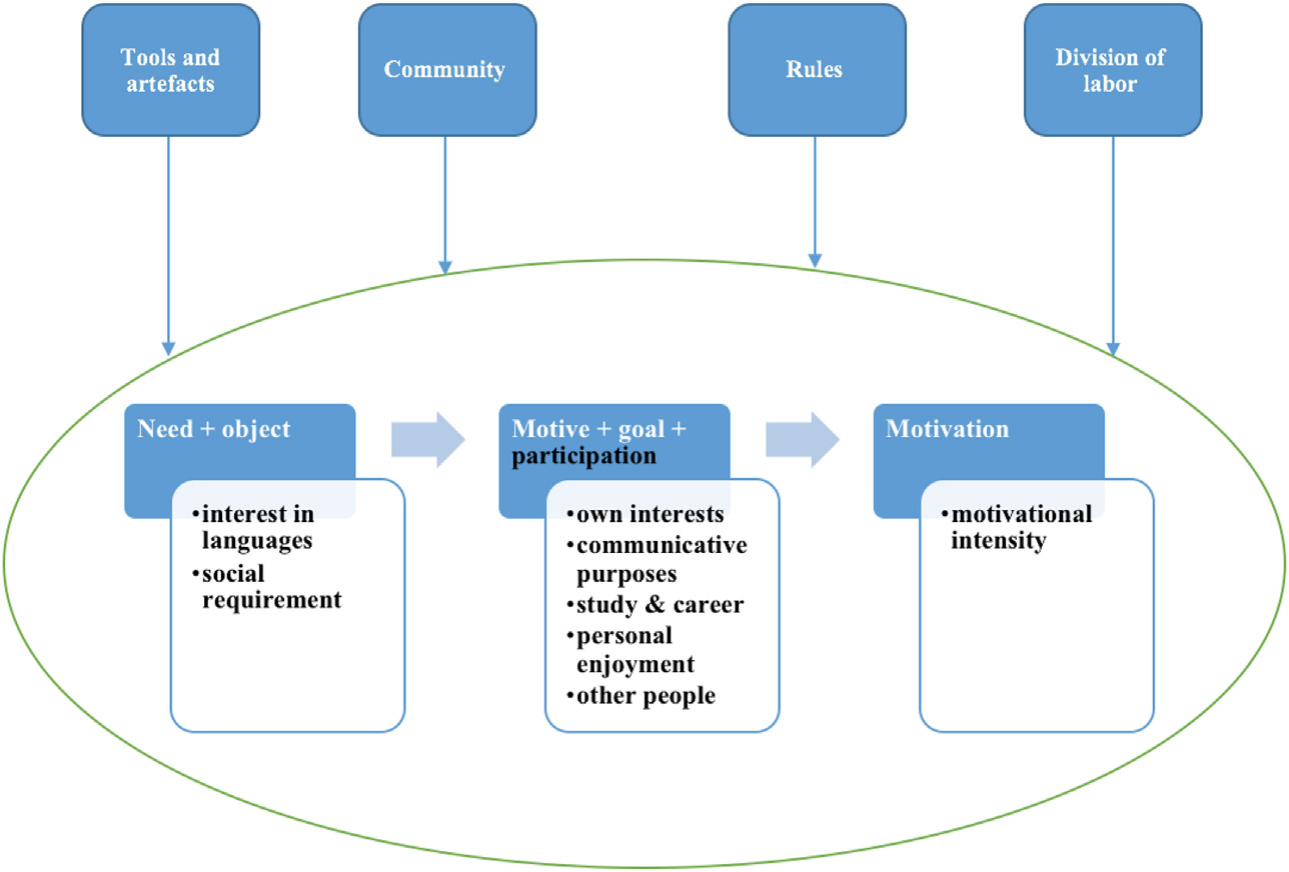
Motivation of the students in the study, reprinted from Second language learning motivation from an activity theory perspective: Longitudinal case studies of Korean ESL students and recent immigrants in Toronto, Kim T. Y., Copyright (2007), with permission from Tae-Young Kim.

It is also notable that the motivational orientations and MI demonstrated by the participants well reflect the development of motivation from activity theory perspective proposed by Kim (2007). When learning English is motivated by biological (i.e., interest in languages) and socially constructed needs (i.e., social requirement of language competence), the needs become motives once directed at an object which embodies students' specific goal. The students had motives and goals, which accommodated the transformation into motivation. That motivation was demonstrated by MI and then specific actions. However, the students lacked participation (colored black in Figure 4) in the community of practice, so their motivation is not fully developed, according to Kim's (2007) framework. In general, elaborating on the activity system by Engeström (1987) with Kim's (2007) interpretation of motivation, we would argue that the participants' needs, objects, motives, goals, participation, and ultimately, motivation are all influenced either positively or negatively by the components of the activity system including the tools, the community, the rule, and the division of labor (Figure 4). For instance, the wide availability of communications with foreigners who speak English, as stated by many students, would exert a positive impact on their participation and finally on their motivation. The explanations above enabled us a lot to provide pedagogical implications in the following part.

## 7 Conclusions

This study investigated the motivation and desire to learn English among a sample of non-English-major students in Vietnam based on the activity theory perspective. The participants included 1,565 students with at least one semester of university-level English, of whom 13 participated in the semi-structured interviews. The findings demonstrated that the students had higher IM than EM, a strong desire to learn English, and a willingness to take opportunities to improve their English proficiency. Moreover, they not only acknowledged the importance of learning English for their academic studies and future careers, but they also learned the language for personal enjoyment, for communicative purposes and because of other people. This study also indicated a strong positive correlation between IM and MI.

In this study, there are two limitations worth noting. First, the scales and interviews did not cover many other aspects regarding the motivation and desire to learn English. Thus, future research should broaden the range of aspects and issues, such as motivation or demotivation, so that more high-quality findings can be produced. Second, there are individual variables, such as age, English proficiency, and attitude, which should be taken into account. Hence, future studies should delve into these variables.

Overall, the present study makes two main contributions to the ELT field and to the literature on motivation. First, academically, since it fills the gap in the research on motivation in a Vietnamese context, it can serve as a source of reference on the motivation to learn English in Vietnam. The study brought an insert to the extensive literature on motivation to learn languages from different perspectives. Second, pedagogically, various stakeholders, such as EFL educators, decision-makers, and curriculum developers, can take this study's findings into consideration to understand more about their students in terms of psychological issues and to design appropriate programs that can increase their learning motivation.

Finally, according to activity theory, these stakeholders include communities based on rules and the division of labor, which are closely related to the subject and object. As a result, what the stakeholders do actually impacts (either positively or negatively) students' motivation. In other words, tension among the elements in the activity system should not be generated and English tertiary education must be supported to develop students’ motivation and to unlock their potential. For instance, since the students in this study were highly motivated to learn English, EFL educators could make the syllabi less examination-oriented and more flexible to encourage more learning activities. Besides, English tertiary education must shift from linguistic and examination-oriented content to English for career development and communicative purposes (see more in Bui, T. Nguyen, & A. Nguyen, 2018) to promote the two types of motivation among non-English majors. Moreover, interactions with selected virtual sites and foreigners should be included in language programmes to facilitate authenticity and participation in the language practice community. With technological advancements, many young people all over the world engage with online communities to learn English that are proven to promote self-regulated learning and facilitate effective learning (Lan et al., 2020). Hence, although participating in such communities have not been widely known and spread, language instructors had better get to know them and involve students in the communities as a way to help enhance their motivation through intercultural experience (see more in Nguyen et al., 2020; Mai et al., 2020).

## Declarations

### Author contribution statement

Son Van Nguyen: Conceived and designed the experiments; Performed the experiments; Analyzed and interpreted the data; Contributed reagents, materials, analysis tools or data; Wrote the paper.

Anita Habók: Conceived and designed the experiments; Analyzed and interpreted the data; Contributed reagents, materials, analysis tools or data.

### Funding statement

This work was supported by the University of Szeged Open Access Fund (Grant number: 4794).

### Data availability statement

The data that has been used is confidential.

### Declaration of interests statement

The authors declare no conflict of interest.

### Additional information

No additional information is available for this paper.
